# Clinical study of transjugular intrahepatic portosystemic shunt combined with AngioJet thrombectomy for acute portal vein thrombosis in non-cirrhosis

**DOI:** 10.1097/MD.0000000000024465

**Published:** 2021-02-12

**Authors:** Zhaonan Li, Wenguang Zhang, De-Chao Jiao, Xueliang Zhou, Pengli Zhou, Guangyan Si, Xinwei Han

**Affiliations:** a Department of Interventional Radiology, First Affiliated Hospital of Zhengzhou University, Zhengzhou; b Department of Interventional Radiology, the Affiliated Hospital of Traditional Chinese Medicine of Southwest Medical University, Luzhou, China.

**Keywords:** portal vein thrombosis, thrombectomy, thrombolysis, transjugular intrahepatic portosystemic shunt

## Abstract

To evaluate the outcomes of the transjugular intrahepatic portosystemic shunt (TIPS) combined with AngioJet thrombectomy in patients with noncirrhotic acute portal vein (PV) thrombosis.

Retrospective analysis from January 2014 to March 2017, 23 patients underwent TIPS combined with AngioJet thrombectomy for acute PV thrombosis in noncirrhosis. The rates of technical success, the patency of the PV, liver function changes, and complications were evaluated.

Twenty-three patients underwent combined treatment, with a technical success rate of 100%. Twenty-four hours after treatment, PV thrombosis grade was improved significantly (*P* = .001). Before and after treatment, Albumin (gm/dl), aspartate transaminase (IU/l), alanine transaminase (IU/l), and platelets (10^9^/L) were all significantly improved (*P* < .05). Minor complications include hematoma, hematuria, and hepatic encephalopathy. After 1 week of treatment, computed tomography scan revealed 8.7% (2/23) cases of hepatic envelope hematoma (thickness less than 2 cm). Hemoglobinuria occurred in 18/23 (78.3%) patients after treatment and returned to normal within 1 to 2 days. Two patients 2/23 (8.7%) had transient grade I encephalopathy after TIPS. The 1-year overall survival rate was 100% (23/23). No major complications during treatment in all patients

AngioJet thrombectomy via TIPS has a favorable short-term effect in clearing thrombus and alleviating symptoms in diffuse acute PVT. The long-term efficacy of this treatment needs to be further studied.

## 1 Introduction

Acute portal vein thrombosis (aPVTs) is rare in noncirrhotic patients, with approximately 0.7 per 100,000 people per year.^[1,2]^ Many aPVTs are still asymptomatic but serious, painful clinical courses have been reported with a mortality rate of up to 75%.^[3]^ For acute PVT, As recommended by the European Association for the Study of Liver (EASL) and the American Association for the Study of Liver, the current mainstay of therapy for aPVTs is anticoagulation with unfractionated heparin or low molecular weight heparin (LMWH), in the absence of contraindications.^[4,5]^ However, 38% of cases can treat aPVTs with anticoagulant monotherapy, but less likely to succeed in chronic and severe cases^[6,7]^ especially in young patients with the severe mesenteric vein, which should be treated more aggressively to prevent intestinal ischemia. The effective therapeutic option has been achieved with Endovascular, catheter-directed thrombolytic therapy, as reported by Lopera.^[8]^ Furthermore, Kim et al^[9]^ also reported similar conclusions, which are currently used in the PVT treatment guidelines.^[10]^ In theory, aPVTs thrombectomy allows for more rapid recanalization of the portal vein (PV) and superior mesenteric vein (SMV). This study was evaluated the clinical efficacy of transjugular intrahepatic portosystemic shunt (TIPS) combined with sequential thrombectomy device AngioJet (Boston Scientific, Natick, MA) in the treatment of patients with non-cirrhotic aPVTs.

## 2 Materials and methods

### 2.1 Patients

From January 2014 to March 2017, all patients with acute PVT treated at the Department of Interventional radiology in the First Affiliated Hospital of Zhengzhou University were enrolled. All patients provided informed consent before admission. The ethics committee approved our research program. The inclusion criteria were as follows:

1. Patients with noncirrhotic PV thrombosis;
2. clear thrombosis in the PV system as seen through Doppler ultrasound, computed tomography angiography (CTA), and/or magnetic resonance angiography;
3. onset of acute thrombosis within 1 week;
4. continued abdominal pain or abdominal distension.

The exclusion criteria were as follows:

1. refusing interventional therapy or follow-up;
2. previous TIPS or thrombolytic therapy;
3. severe cardiac or lung disease;
4. aPVTs after liver transplantation;
5. malignant tumor; and
6. contraindications to anticoagulation therapy.

### 2.2 Evaluation of PVT status

Doppler ultrasound, CTA, or magnetic resonance angiography for assessing thrombosis in terms of location and severity. The PVT location was divided into the main portal vein (MPV), splenic vein (SV), and SMV. The PVT severity was divided into 4 levels: grade 0 (thrombus deficiency), grade I (MPV thrombus <50% or only SMV and SV thrombus existed), grade II (MPV thrombus accounted for 50%–100%), and grade III (complete blocking or cavernous transformation of the PV)

### 2.3 Intervention

**Figure 1:**
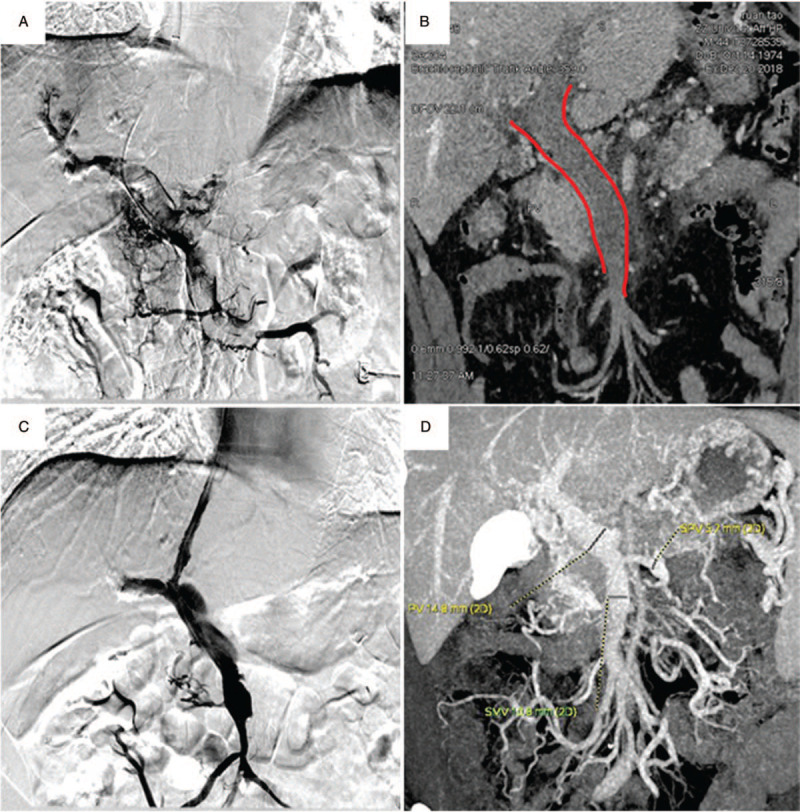
Establishing a shunt combined thrombectomy device via the TIPSS route. A 52-year-old man hospitalized with portal hypertension and diagnosed with protein S insufficiency (A) Acute thrombosis portal vein, splenic vein and superior mesenteric vein – diagnostic contrast agent injected into the abdominal vein; (B) Preoperative enhanced CT showed extensive portal vein thrombosis, Red line; (C) Controlled angiography during lysis- patency of superior mesenteric vein and main portal vein; (D) Enhanced CT angiography revealed superior mesenteric vein and portal vein trunk without thrombus attachment.

**Figure 2:**
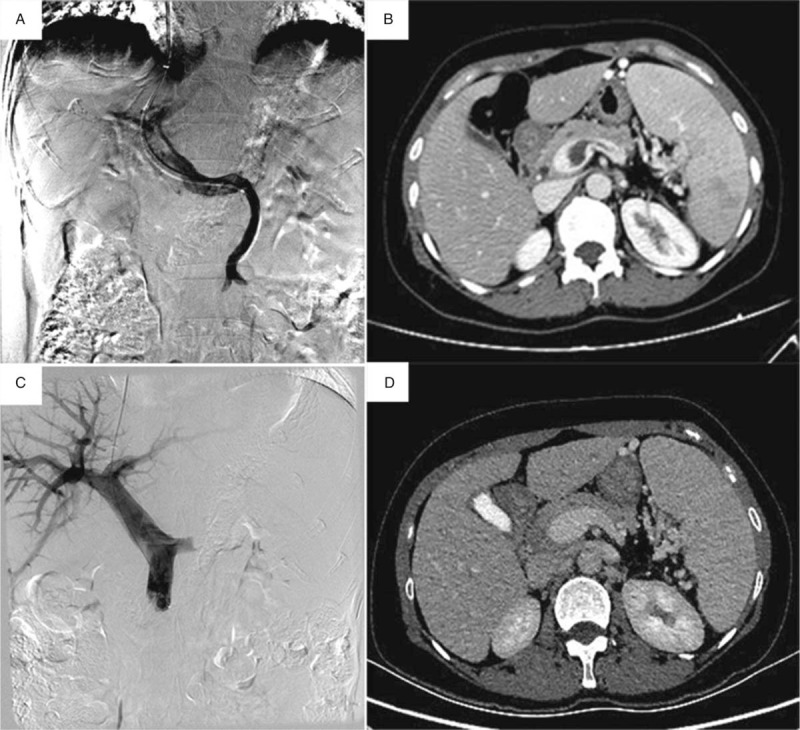
AngioJet thrombectomy through the portal shunt only. A 65-year-old man hospitalized with acute gastrointestinal bleeding and diagnosed with essential thrombocythemia (A/B) Acute thrombosis portal vein diagnostic contrast agent injected into the abdominal vein; (C/D) Angiography shows patency of the portal vein.

In all 23 patients, a transjugular approach was used. The transhepatic puncture was performed under ultrasound guidance. A hydrophilic wire (Terumo, Tokyo, Japan) was threaded through the puncture cannula and a 5-Fr vertebral catheter (Cook Medical, Bloomington, USA) was subsequently advanced into the PV, the lineal vein, and the SMV show the extent of thrombosis. Mechanical thrombectomy was then performed on the PV and SMV by a guidewire placed into the xpeedior thrombus aspiration catheter. The patient was treated with urokinase 300,000 u (dissolved into 100 ml of normal saline) and the aspiration catheter was subjected to thrombus aspiration at a rate of 1 mm/s along the guidewire. Despite the significant reduction in thrombus burden, venous blood flow has not been fully restored, so we inserted a porous infusion catheter along the SMV and performed 24-hour thrombolytic therapy with a high dose of urokinase (100,000 units/hr). In addition, 5000 IU of LMWH was injected twice a day, and warfarin was administered after treatment at an initial dose of 5 mg. Thrombolysis time depended on the improvement of the patient's clinical symptoms, decrease in D-dimer, and imaging data. The LMWH was discontinued when the international normalized ratio was maintained at 2.0 to 3.0, and warfarin therapy for at least 6 months followed by 100 mg/d aspirin monotherapy for a lifetime. The treatment information is shown in (Figs. 1 and 2)

### 2.4 Outcome measurements

The outcome measure was the change in PV patency status, which was evaluated by CTA or Doppler ultrasound. Changes in PV patency status were defined as follows:

1. recanalization, with complete disappearance or reconstruction of cavernous transformation;
2. improved, with recanalization improvement from grade III or II to grade II or I, or disappearance of an SMV or SV thrombus;
3. stable; and
4. worsened, with worsening from grade I or II to grade II or III, progression of thrombus to cavernous transformation, or formation of an SMV or SV thrombus.

### 2.5 Follow-up

All patients were followed at 12 months after the treatment. Laboratory testing (coagulation function, routine blood test) and CTA/Doppler ultrasound were performed at each arranged follow-up visit, or whenever clinically required (eg, for ascites, black stools, or abdominal pain). During follow-up, no blind method was adopted for patients taking warfarin.

### 2.6 Statistical analysis

Statistical analyses were performed using SPSS version 22.0 software (SPSS Inc., Chicago, IL). *P*-value <.05 defined as statistically significant. Data are presented as the mean ± SD or expressed as a percentage. With a paired-sample *t-*test and Fisher exact test for data measurement,

## 3 Results

### 3.1 Patient characteristics

This study retrospectively analyzed the condition of 23 patients with noncirrhotic acute PVT who received TIPS combined with AngioJet thrombectomy from January 2014 to March 2017, including essential thrombocythemia in 6/23 (26.1%), protein S insufficiency 4/23 (17.4%), oral contraceptives 3/23 (13.0%), JAK2 mutation 3/23 (13.0%), antiphospholipid syndrome 2/23 (8.7%) cases whilst 5/23 (21.7%) cases reported acute PVT without further specification. MPV thrombus grade I was reported in 3/23 cases (13.0%), grade II in 11/23 cases (47.8%), Grade III in 9/23 cases (39.1%) whilst 9/23 cases (39.1%) were SMV thrombus and 8/23 cases (34.8%) were SV thrombus. The characteristics of the combined group were in (Table 1).

**Table 1:** Baseline characteristics.

Characteristic	Value
Age	49.8 ± 12.1
Male sex	17
Etiology	
essential thrombocythemia	6
protein S insufficiency	4
oral contraceptives	3
JAK2 mutation	3
antiphospholipid syndrome	2
unknown	5
Child-Pugh class	
A	4
B	15
C	4
Child-Pugh score	8.6 ± 2.9
MELD score	9.1 ± 4.6
Past gastrointestinal bleeding	13
MPV thrombus	
Grade I	3
Grade II	11
Grade III	9
SMV thrombus	9
SV thrombus	8

Data are mean plus or minus standard deviation. MELD = model for end-stage liver disease, MPV = main portal vein, SMV = superior mesenteric vein, SV = splenic vein.

### 3.2 Changes in liver function and PV thrombosis

Before and after treatment, ALB (gm/dl), aspartate transaminase (IU/l), alanine transaminase (IU/l), and platelets (10^9^/L) were all remarkably improved (*P* < .05) (Table 2). Fourteen out of twenty-three patients underwent sequential AngioJet thrombectomy through the portal shunt only, and 9/23 patients underwent sequential AngioJet thrombectomy via TIPS route for implanted stent as adjuvant therapy. Twenty-four hours after treatment, PV thrombosis grade was improved significantly (*P* = .001) (Table 3). The number of patients with portal recanalization at 3, 6, and 12 months was 8/23 (34.8%), 7/23 (30.4%), and 4/23 (17.4%), respectively (*P* = .497). The number of people who showed improvement was 10/23 (43.5%), 9/23 (39.1%), 7/23 (30.4%), respectively (*P* = .743). while, patients who appeared stable was 2/23 (8.7%), 4/23 (17.4%), and 7/23 (30.4%) at 3, 6, and 12 months after treatment, respectively (*P* = .201) (Table 4).

**Table 2:** Changes in laboratory indicators after 1month of treatment.

	Before treatment^∗^	After 1 month of treatment^∗^	*P* value
ALB (gm/dl)	35.8 ± 5.4	39.9 ± 4.2	.002
TB (mg/dl)	15.2 ± 3.4	12.4 ± 4.1	.013
AST (IU/l)	71.3 ± 11.5	47 ± 18.3	.000
ALT (IU/l)	52 ± 9.3	44.7 ± 10.9	.001
INR	1.12 ± 0.4	2.5 ± 0.6	.755
PLT (10^9^/L)	355 ± 105.3	283 ± 98.7	.002

ALB = albumin, ALT = alanine transaminase, AST = aspartate transaminase, INR = international normalized ratio, PLT = platelets, TB = total bilirubin.

∗ Data expressed as mean ± SD.

**Table 3:** Changes in portal vein thrombosis after 24 hours of treatment.

	Before	After	*P* value
MPV thrombus			.001
Grade 0	0	6	
Grade I	3	10	
Grade II	11	5	
Grade III	9	2	
SMV thrombus	9	3	.091
SV thrombus	8	2	.071

MPV = main portal vein; SMV = superior mesenteric vein; SV = splenic vein; Data are numbers of patients.

**Table 4:** Changes in portal vein thrombosis during follow-up.

	Portal vein (*n* = 23)			*P* value
Time (mo)	3	6	12	
Recanalization	8	7	4	.497
Improved	10	9	7	.743
Stable	2	4	7	.201
Worse	3	3	5	.772

Recanalization = complete disappearance or reconstruction of cavernous transformation; improved = recanalization improvement from grade III or II to grade II or I, or disappearance of an SMV or SV thrombus; stable = No significant changes; worsened = worsening from grade I or II to grade II or III, progression of thrombus to cavernous transformation, or formation of an SMV or SV thrombus. Data are numbers of patients.

### 3.3 Complications

The patients were followed up for an average of 12 months. The 1-year overall survival rate was 100% (23/23). Minor complications include hematoma, hematuria, and hepatic encephalopathy. After 1 week of treatment, CT scan revealed 8.7% (2/23) cases of hepatic envelope hematoma (thickness less than 2 cm). Hemoglobinuria occurred in 18/23 (78.3%) patients after treatment and returned to normal within 1 to 2 days. Two patients 2/23 (8.7%) had transient grade I encephalopathy after TIPS, and all of them were relieved after medical combination therapy.

## 4 Discussion

In noncirrhotic patients, aPVTs is a relatively rare disease and commonly caused by myeloproliferative diseases, congenital coagulopathies, hypercoagulable states (insufficiency of antithrombin III, protein C, protein S, etc), or oral contraceptives use.^[1,11]^ Severe aPVTs leads to intestinal ischemia or infarction with perforation and peritonitis, which is the most troubling immediate complications. The consensus document of the Baveno VI conference included revised treatment recommendations for noncirrhotic PVT: initial therapy is identical to that of cirrhotic PVT, with immediate commencement of low-molecular-weight heparin followed by oral anticoagulant therapy.^[10]^ However, there is no interdisciplinary consensus for the management of acute PVT in the case of treatment failure with standard anticoagulation and conservative treatment does not directly eliminate obstruction due to thrombosis. Maldonado et al^[12]^ reported that patients with anticoagulant therapy alone had an extremely high incidence of portal hypertension during follow-up. Therefore, more aggressive and effective treatment strategies may be required.

Traditional thrombolysis and anticoagulant therapy cannot directly eliminate obstruction caused by thrombosis.^[13,14]^ Compared with conservative therapy, endovascular techniques can reconstruct blood vessels as soon as possible, reducing intestinal ischemia, intestinal necrosis, and complications caused by portal hypertension, are the promising treatment of aPVTs. Liu et al^[15]^ showed the effect of direct thrombolysis is superior to indirect treatment. Injecting thrombolytic agents directly into PV-SMV thrombus can significantly increase local drug concentrations, reduce systemic thrombolytic doses and bleeding complications. Recently, intravascular endovascular therapy mainly includes catheter thrombolysis or mechanical thrombectomy, and AngioJet assisted mechanical thrombectomy could be used in combination with these 2 treatment modes. Its therapy has the advantages of minimally invasive, low consumption of thrombolytic agents, no major bleeding from thrombolysis, etc. Currently, the path of mechanical thrombolysis used frequently includes a transcutaneous transhepatic or transjugular route.

For patients with complications of portal hypertension, it is more suitable for the combination of TIPS and AngioJet, to date it is not an established therapy option. After the shunt is established, the risk of thrombosis could be reduced due to an increase in blood flow velocity of the PV. Kuetting et al^[16]^ reported 4 patients with acute and acute-on-chronic (1 case) acute PVT underwent initial AngioJet thrombectomy followed by TIPSS with Viatorr stent-graft and directed intravenous thrombolysis along the shunt. The technical success rate was 100% and 75% of cases have achieved therapeutic success. This report indicates that combination therapy can significantly reduce the thrombus burden in acute patients, and PV can be recanalized in all However, in acute-on-chronic cases, the initially opened PV was re-occluded after 10 days of combined surgery. Among 9 patients reported by Wolter et al,^[17]^ 4 were noncirrhotic extensive PV thrombosis, and the technical success rate also was 100%. It seems to be of utmost importance to not only reduce the thrombotic material but to establish sufficient inflow and outflow by TIPS and simultaneous multicatheter thrombolysis. In our study, 23 patients underwent combined treatment, with a technical success rate of 100%. Twenty-four hours after combined treatment, PV thrombosis grade was improved significantly (*P* = .001). The 1-year overall survival rate was 100% (23/23). AngioJet thrombectomy via TIPS in this study has a good short-term effect on clearing thrombus and alleviating symptoms in acute PVT.

Our research has some limitations. First, due to the scarcity of the disease, the number of patients enrolled in this group is limited. Second, this is a retrospective study, and the short follow-up time may exaggerate the actual results. Finally, the combination of TIPS and AngioJet thrombectomy is not a standard treatment for aPVTs. Therefore, a large number of randomized controlled trials are needed to validate this result. In conclusion, AngioJet thrombectomy via TIPSS for aPVTs in noncirrhosis has a favorable short-term effect in clearing thrombus and alleviating symptoms in diffuse aPVTs.

## Acknowledgments

We would like to thank the patients and all employees of the First Affiliated Hospital of Zhengzhou University for interventional radiology. Thanks to Ms. Chen Xinyue from CT collaboration NE Asia, Siemens Healthcare, Beijing, China, she contributed strong technical support as well as language polish to this article.

## Author contributions

**Conceptualization:** Zhaonan Li, Xinwei Han.

**Data curation:** Dechao Jiao.

**Formal analysis:** Dechao Jiao.

**Funding acquisition:** Dechao Jiao.

**Investigation:** Wenguang Zhang, Guangyan Si.

**Methodology:** Wenguang Zhang, Pengli Zhou.

**Project administration:** Pengli Zhou.

**Validation:** Xueliang Zhang.

**Writing – original draft:** Zhaonan Li.

**Writing – review & editing:** Xueliang Zhou.

## Abbreviations

Abbreviations: aPVTs = acute portal vein thrombosis, CTA = computed tomography angiography, LMWH = low molecular weight heparin, MPV = main portal vein, PV = portal vein, SMV = superior mesenteric vein, SV = Splenic vein, TB = total bilirubin, TIPS = transjugular intrahepatic portosystemic shunt.
